# Assessing the knowledge and skill of vaccination staff at Adult Vaccination Counters for COVID-19 vaccines: Simulated client method

**DOI:** 10.1371/journal.pone.0261286

**Published:** 2021-12-23

**Authors:** Wajiha Qamar, Mehran Qayum, Naveed Sadiq

**Affiliations:** 1 Bacha Khan College of Dentistry, Mardan, Pakistan; 2 Oxford Policy Management (OPM), Peshawar, Pakistan; 3 Institute of Public Health & Social Sciences, Khyber Medical University, Peshawar, Pakistan; Middle East Liver Diseases (MELD) Center, Tehran, Iran, ISLAMIC REPUBLIC OF IRAN

## Abstract

The Government of Pakistan has established Adult Vaccination Counters (AVCs) to immunize general population with COVID-19 vaccine. Different brands of COVID-19 vaccines have different protocols. It is important that the knowledge and skills of the vaccination staff at AVCs should be accurate. To assess this, a cross-sectional study was conducted in all 15 AVCs at Khyber Pakhtunkhwa’s provincial capital in May 2021, using the simulated client approach. Structured open-ended and simulated scenario-based questions were used to collect data from the vaccination staff of AVCs. This study showed that 53.3% of the AVCs had at most three out of four brands of COVID-19 vaccines. 60% of the AVCs did not have the mechanism to track client’s vaccine first dose, date, and brand. Only 66.7% of the AVCs had a complete knowledge of all the available vaccines. 86.7% and 80% of the AVCs knew the correct duration and administration of the same brand of COVID-19 vaccine’s second dose respectively. At the client’s end, 6.7% were aware about the brand of administered COVID-19 vaccine. 46.7% were advised about the date of the second shot of vaccination. Only 13.3% of the clients were informed about the procedure of getting an official vaccination certificate. It was concluded that the knowledge and skill of the vaccination staff at AVCs is inadequate. Every vaccine has a different protocol in terms of number of doses and duration. AVCs must have a tracking system to inoculate the second dose with the same brand as the first dose. There is a need for rigorous monitoring and training of the COVID-19 vaccination staff on various protocols of vaccine to prevent losing public’s trust.

## Introduction

As COVID-19 pandemic is sweeping the globe, the World Health Organization (WHO) and partners are working together to produce and deploy safe and effective vaccines, monitoring the pandemic, advising on urgent measures, and supplying essential medical resources to those in need [1]. The vaccines for COVID-19 are being produced at a breakneck pace to control the pandemic [2]. The efforts for COVID-19 vaccine development are directed towards client safety and efficacy. The WHO has approved a total of seven vaccines so far, that are safe and reliable and can help people from becoming chronically ill or dying from COVID-19 [3]. These eight WHO approved vaccines include: Moderna, Pfizer/BioNTech, Janssen (Johnson & Johnson), Oxford/AstraZeneca (AZ) vaccine, Covishield, Sinopharm (SP), Sinovac (SV), and Covaxin [3]. These COVID-19 vaccines provide a high level of protection against being chronically ill or dying from the disease, despite the fact that no vaccine is 100% effective [1]. At least 206 countries and territories have administered more than 1 billion doses of a COVID-19 vaccine [4].

Pakistan has experienced three waves of COVID-19 and is currently handling the fourth Delta-variant wave. The Government of Pakistan (GoP) has established the National Command and Operation Center (NCOC) to synergize and articulate unified national efforts against COVID-19. NCOC reported 1,152,481cases as of August 30^th^, 2021, with 25,604deaths in the country [5, 6]. The GoP has approved the administration of four WHO approved COVID-19 vaccines to the general public, namely SP, SV, AZ as well as a non-approved WHO vaccine CanSino Bio (CSB), for emergency use in the country [7].

Adult Vaccination Counters (AVCs) have been set up in nearly all of the provincial districts. These AVCs have been staffed with relevant trained personnel to smoothen the uptake of COVID-19 vaccination [6]. Moreover, the Pakistan’s Ministry of National Health Services, Regulation and Coordination has shared comprehensive guidelines for the establishment of AVCs in every district. According to national guidelines, every AVC must have a computer, printer, android phone, internet access, and a constant power supply to ensure a smooth immunization procedure [8]. The District Health Monitoring Teams have been mandated to oversee the vaccination procedure at the AVCs [8]. COVID-19 vaccination is being carried out at 582 AVCs across Pakistan, covering approximately all of the country’s districts [8]. Around 174 AVCs in Khyber Pakhtunkhwa are providing COVID-19 vaccinations.

According to NCOC statistics, 16,171,867 persons were fully vaccinated as of 30 August, 2021, for a total of 53,314,628 doses [6, 7]. While other South Asian countries ramp up their immunization programs, GoP is putting all its efforts towards the mass immunization to achieve herd immunity against COVID-19 [9]. Several communication strategies have been used in Pakistan to encourage people to be vaccinated against COVID-19. Although the campaigns have had some success, more comprehensive and coordinated efforts are required to involve the population in the vaccination push in order to address attitudinal barriers in Pakistan. As per national guidelines, there should be a designated vaccination staff at each of the AVCs. Therefore, the knowledge regarding the vaccines should be up to date as per the national guidelines for each vaccine. This is important because vaccine hesitancy has been found to be very high in the country [10]. With poor knowledge and skill of the vaccination staff, the vast immunization efforts of the country may go in vain. In this regard, we conducted this study with the goal of determining the knowledge and skill of the vaccination staff regarding COVID-19 vaccines under which the AVCs are operating at the provincial capital of Khyber Pakhtunkhwa.

## Methods

This is a descriptive cross-sectional study that was carried out in May 2021 using simulated client method [11]. As there was no funding available to support this study for the whole province, therefore, it was conducted only in Peshawar, the provincial capital of the Khyber Pakhtunkhwa province. At the time of the study, 15 AVCs were established in Peshawar’s public sector health facilities, administering COVID-19 vaccines. The questionnaire assessed the areas such as usage of automated technologies for record keeping, tracking, and service providers’ understanding of vaccination, its dosages, administration, and client follow-up as detailed in the guidelines.

Instead of approaching the vaccination staff with the traditional approach of interviewing and to avoid Hawthorne effect [12], the researcher, using the simulated client method, disguised as clients approached the vaccination staff at each of the AVCs. A total of two researchers/authors were trained to be disguised as clients. Two AVCs were used to pilot test the scenarios and thus the questionnaire. Some adjustments were made in both the simulated scenarios and the questionnaire. These included the tool’s logical sequence, additional questions to improve the capture of required data, and the removal of unnecessary repetitions. The ethical permission to conduct this study was obtained from the Khyber Medical University, Pakistan (approval number: DIR/KMU-EB/AK/00065).

The researcher asked questions or observed, as a client, on the vaccination centers and the knowledge of the vaccination staff at each of the vaccination centers. For the vaccination centers, questions on the type and number of various types of vaccines were asked. Furthermore, information on tracking system for the first dose, and cadre of health personnel administering the vaccine and entering the record in the computer system (where available) were observed and/or acquired from the staff at each of the vaccination center. Lastly, information on the last client exiting the health facility was also captured to assess if the client knows the name of the vaccine administered, period after which the client will receive the second dose, and the procedure to obtain the official vaccination certificate. Only one real client was chosen at random at each of the AVC, after exiting, to assess if they were informed on the vaccine administered. The last real client exiting the health facility was approached by the simulated client, seeking information from the real client like it happens in the real world. More real clients were not engaged as this would have raised a suspicion for the simulated client as an inspector or a data gatherer.

The knowledge of the vaccination staff was tested on the brands of the vaccine available at their respective AVCs. To gauge this, a collective and complete knowledge of all the available vaccines in terms of their knowledge of correct doses, duration between the first and the second dose, and whether another brand of vaccine could/could not be administered on the second visit was assessed from the vaccination staff of the AVCs. Additionally, the correct number of doses, the correct duration of the second dose (if applicable), the knowledge of the vaccination staff that second dose of the same vaccine brand should be administered as of the first dose, and the complete knowledge of the individual vaccines available at their respective AVCs was also assessed in this study.

To obtain this information, the researchers, like any other client, seeked information for themselves and/or for their families from the vaccination staff of the AVCs, as per designed scenario. The researcher practiced as a client in the office settings before being deployed in the field. The scenarios were designed to gather information on the above variables. The scenarios and the questionnaire designed for this study could be found in S1 Questionnaire. The gathered information from the vaccination staff at each of the AVCs, using scenario-based questions, was immediately entered in to the customized questionnaire as the researcher exited the AVC.

## Results

The results were divided into two categories. The first category of results were related to the AVCs and the skills of the vaccination staff (Table 1) while the second category of results pertained to the knowledge of the vaccination staff at the AVCs (Table 2).

**Table 1:** Information about the Adult Vaccination Counters (N = 15).

Variables		Percentage
Types of vaccines present	Sinovac	80
	Sinopharm	46.7
	CanSino Bio	13.3
	AstraZeneca	93.3
Number of various types of vaccines available at the health facility	1	13.3
	2	33.3
	3	53.3
Vaccination counter has a system to track the client’s first dose	Yes	40
Health Personnel Administering Vaccine	Medical Technician	80
	Lady Health Visitor/Nurse	13.3
	Doctor	6.7
Health Personnel Entering Record into Computer Systems	Medical Technician	66.7
	Receptionist	33.3
Check with the last client to see if they were informed about the:	Name of the vaccine administered	6.7
	Period after which the client will receive the second dose	46.7
	Procedure to obtain official vaccine certificate	13.3

**Table 2:** Knowledge of the vaccination staff on available COVID-19 vaccines at the AVCs.

		Percentage
Vaccination staff aware of the brands of vaccines available at the AVC	Yes	93.3
Vaccination staff has a complete knowledge of all available vaccines	Yes	66.7
Vaccination staff knows correct number of doses of all available vaccines	Yes	100
Vaccination staff knows the correct duration of the second vaccine dose	Yes	86.7
Vaccination staff knows that second dose of the same brand vaccine should be administered as of the first dose	Yes	80
Vaccination staff has a complete knowledge of Sinovac	Yes	75
Vaccination staff has a complete knowledge of Sinopharm	Yes	71.4
Vaccination staff has a complete knowledge of CanSino Bio	Yes	100
Vaccination staff has a complete knowledge of AstraZeneca	Yes	85.7

Table 1 shows that almost nine in ten AVCs had AZ available with them. There were a total of 15 AVCs in the Peshawar District at the time of study. Out of these, more than half of the AVCs had three types of COVID-19 vaccines available in their facility. Two in five AVCs knew the mechanism of tracking the client’s first vaccine dosage date and brand of the vaccine. There were two separate stages of vaccination at the AVCs, administration and recording of the administered vaccine into the governmental database. At each of the stages, there were different healthcare personnel involved in the process. Majority of the vaccines were administered and recorded in the database by the Medical Technicians (80% and 66.7% respectively). Very few clients (6.7%) were informed of the brand of COVID-19 vaccine. A total of 15 real clients were approached, one from each AVC. Less than half of the clients (46.7%) were informed of the period of the second dose of vaccine (if applicable) following their first jab. Almost one in ten clients were informed on the process of obtaining official vaccine certificate.

Table 2 shows that the majority of the AVCs were aware of the brands of vaccines available at their counters. Two-third of the AVCs had a complete knowledge of the available vaccines. The AVCs personnel knew about the correct number of doses of each available brand of vaccine. Almost nine in ten AVCs personnel knew the correct duration of the second dose of the administered vaccine. The vaccination staff in four out of five AVCs knew that the second dose of the same brand vaccine should be administered.

## Discussion

This study was conducted with the aim to determine the knowledge and skill of the vaccination staff regarding COVID-19 vaccines under which the AVCs are operating at the provincial capital of Khyber Pakhtunkhwa. The findings of this study indicate that at least at one-third of the AVCs in the provincial capital of Khyber Pakhtunkhwa don’t have a complete knowledge of COVID-19 immunization guidelines, e.g. a quarter or less of the AVCs are deficient in information on one or more aspects on commonly used vaccines like SV and SP. CSB is the least available vaccine but the vaccination staff’s knowledge on CSB was found to be perfect in terms of total number of doses required to achieve immunity. The primary reason for this could be that CSB requires only a single dose of vaccine, therefore, the protocols for CSB were simple and easy to comprehend. The second vaccine for which the vaccination staff’s knowledge was relatively better was AZ as compared to SV and SP. The knowledge about each brand of vaccine is very important. The reason being that vaccines trials have not yet demonstrated any immunization-related adverse drug events if the brands are cross-mixed, thus, it is critical to adhere to the approved vaccine guidelines for now [13].

Regarding knowledge on the second dose of vaccine, one in five did not have the knowledge that the same brand of vaccine should be used on the second dose as on the first dose. The national guidelines as well as the Centers for Disease Control and Prevention guidelines advise not to combine different brands of vaccines unless there are "extraordinary circumstances," such as a lack of the vaccine they received initially due to manufacturing or delivery difficulties [14]. Though, combining vaccine doses of different brands may provide an opportunity to speed vaccine rollouts and, potentially enhance the immunity provided, however, data supporting the appropriateness of this strategy is still lacking in the case of COVID-19 and is under trials [15]. Until the complete findings of these trials are out, health experts are recommending caution [16]. One of the reasons for this caution are the preliminary findings from the Com-COV trial, which compared mixed doses schedules of Pfizer and Oxford-AstraZeneca vaccines, revealing an increase in the incidence of mild to moderate symptoms in individuals who received mixed dosing schedule, indicating heightened reactogenicity [17].

WHO and CDC strongly advocate that all health workers, involved in COVID-19 vaccination, to have appropriate knowledge and skills to guarantee safe and efficient COVID-19 vaccine delivery [18, 19]. Therefore, COVID-19 vaccination staff must be adequately trained. Without proper and effective training, the vaccination campaigns for COVID-19 may be subject to failure as this region has one of the highest refusals when it comes to vaccinations, e.g. polio [20]. A study found that 40% of the healthcare workers in Pakistan are hesitant to get any COVID-19 vaccine [10], even though healthcare workers have relatively more access to medical facilities and engage in more health-seeking behaviors than the non-healthcare workers [21]. Among healthcare workers willing to get any vaccine, 61% opted for Chinese brands (SP & SV) [10]. A common person’s hesitancy to COVID-19 vaccine or even a complete refusal may be further evident if the knowledge and skill of the vaccination staff of the AVCs is inadequate. To overcome this obstacle, vaccines with only single dosage may be deemed as a better option in Pakistan, e.g. CSB. This study has also shown that the vaccination staff had a complete knowledge of CSB, however, the only caveat with CSB is that it is not approved by the WHO yet. In the light of these arguments, it may be recommended that the government should ensure strict adherence of COVID-19 vaccination staff to COVID-19 vaccination protocols/guidelines in case of the availability of multiple brands of COVID-19 vaccines. Alternatively, the government may opt for a single dose COVID-19 vaccine such as CSB to avoid any disaster both at the patient and vaccine management levels and also for boosting public’s trust on COVID-19 vaccines.

Even though the government has set up an online database and verification system that entails a complete record of vaccine brand and date on which it was administered. However, the knowledge about this database was not frequently known among the vaccination staff of most of the AVCs. Moreover, majority of the AVCs did not have a system to track the client’s first dose of vaccine or they did not know a way to figure out what brand of COVID-19 vaccine did the client receive, when, and where. Though the AVCs implementation guidelines require the establishment of information systems at each of the AVCs, e.g. the possession of a functioning computer with internet and constant electric power or alternatively, possession of an android based device linked with a functioning internet to access the databased provided by the Government of Pakistan to track details. This is why the vaccination staff at majority of the AVCs was found to be unaware of the system to track the first dose of vaccine. It would be in the best interest of both the public and the AVCs if a manual vaccination card was also issued to the clients in addition to making an entry in the online database system [8, 14, 22]. This way a double verification system may be established in hard and soft forms. Research has also shown that double entry method is the most effective for preventing and catching errors related to data entry [22]. Though some may critique that urgency and emergency to vaccinate people on mass scale may have withheld the double entry method, however, accuracy and precision in such situations is equally important to further prevent vaccine hesitancy in a country where conspiracy theories are prevalent, polio outbreaks still occur, and there is COVID-19 vaccine hesitancy additionally.

This study is limited by the AVCs of the provincial capital of the province only. But its results are generalizable, at least, to the whole province. The overseeing in the provincial capital is usually stricter than the rest of the cities in the province. Therefore, the COVID-19 vaccine situation in other cities of the province may be judged from the situation in the provincial capital.

## Conclusions

The knowledge and skill of the vaccination staff at AVCs is inadequate. Every vaccine has a different protocol in terms of number of doses and duration. AVCs must have a tracking system to inoculate the second dose with the same brand as the first dose. There is a need for rigorous monitoring and training of the COVID-19 vaccination staff on various protocols of vaccine to prevent losing public’s trust.

## Supporting information

S1 Questionnaire Study tool & its associated scenario. (DOCX)

## References

[1] WHO. COVID-19 advice for the public: Getting vaccinated 2021 [https://www.who.int/emergencies/diseases/novel-coronavirus-2019/covid-19-vaccines/advice.

[2] Lurie Nicole, Saville Melanie, Hatchett Richard, Halton Jane. Developing Covid-19 Vaccines at andemic Speed. New England Journal of Medicine. 2020;382:1969–73. 10.1056/NEJMp2005630 32227757

[3] 7 Vaccines Approved for Use by WHO: McGill COVID19 Vaccine Tracker Team; 2021 [https://covid19.trackvaccines.org/agency/who/.

[4] Pettersson H, Manley B, Hernandez S, McPhillips D. Tracking Covid-19 vaccinations worldwide 21 May 2021 [https://edition.cnn.com/interactive/2021/health/global-covid-vaccinations/.

[5] Health Emergency Dashboard (COVID-19) [Internet]. 2021. https://covid19.who.int/region/emro/country/pk.

[6] NCOC. National Command and Operation Center: Vaccine Stats 30 August 2021 [https://ncoc.gov.pk/#section2.

[7] DRAP. News and Updates—COVID-19 2021 [https://www.dra.gov.pk/Home/covid.

[8] Ministry of National Health Services RC. Guidelines for Adult Vaccination Counters (AVCs) in Pakistan. 28 January 2021.

[9] Khan S. COVID: What’s behind Pakistan’s low vaccination rate? DW. 10 May 2021.

[10] Rehman Khalid, Hakim Muhammad, Arif Nauman, Ul Islam Siraj, Saboor Abdul, Asif Muhammad, et al. COVID-19 vaccine acceptance, barriers and facilitators among healthcare workers in Pakistan. Journal of Infectious Diseases & Therapy. 2021;https://www.omicsonline.org/open-access/covid19-vaccine-acceptance-barriers-and-facilitators-among-healthcare-workers-in-pakistan.pdf.

[11] Madden J M, Quick J D, Ross-Degnan D, Kafle KK. Undercover careseekers: simulated clients in the study of health provider behavior in developing countries Social Science & Medicine. 1997;45(10):1465–82. 10.1016/s0277-9536(97)00076-2 9351137

[12] Fitzpatricka A, Tumlinson K. Strategies for Optimal Implementation of Simulated Clients for Measuring Quality of Care in Low- and Middle-Income Countries. Global Health: Science and Practice. 2017;5(1):108–14. 10.9745/GHSP-D-16-00266 PMC5493448 28126970

[13] Cohen Adam F., van Gerven Joop, Burgos Juan Garcia, de Boer Anthonius, Foucher Ron A. M., Flore H., et al. COVID-19 vaccines: the importance of transparency and fact-based education. British Journal of Clinical Pharmacology. 2020;86(11). doi: 10.1111/bcp.14581 33464636 PMC7576612

[14] CDC. Vaccines & immunizations: CDC; [https://www.cdc.gov/vaccines/covid-19/info-by-product/moderna/moderna-faqs.html.

[15] Parkins K. Covid-19 vaccine mixing: the good, the bad and the uncertain. Clinical Trials Arena. 2021.

[16] Shaw Robert H, Stuart Arabella, Greenland Melanie, Liu Xinxue, Nguyen Van-Tam Jonathan S, Snape Matthew D, et al. Heterologous prime-boost COVID-19 vaccination: initial reactogenicity data. Lancet. 29 May 2021;397:2043–5. doi: 10.1016/S0140-6736(21)01115-6 33991480 PMC8115940

[17] Shaw Robert H, Stuart Arabella, Greenland Melanie, Liu Xinxue, Nguyen Van-Tam Jonathan S, Snape Matthew D, et al. Heterologous prime-boost COVID-19 vaccination: initial reactogenicity data. The Lancet. 12 May 2021;397(10289). doi: 10.1016/S0140-6736(21)01115-6 33991480 PMC8115940

[18] WHO. COVID-19 vaccination training for health worker 2021 [https://openwho.org/courses/covid-19-vaccination-healthworkers-en.

[19] CDC. COVID-19 Vaccination Training Programs and Reference Materials for Healthcare Professionals. 07 April 2021.

[20] Hussain Shoaib Fahad, Boyle Peter, Patel Preeti, Sullivan R. Eradicating polio in Pakistan: an analysis of the challenges and solutions to this security and health issue. Globalization and Health volume 2016;12(63). doi: 10.1186/s12992-016-0195-3 27729081 PMC5059991

[21] Bana Shazia, Yakoob Javed, Jivany Nourin, Faisal Asima, Jawed H, Awan S. Understanding Health Seeking Behavior Of Health Care Professionals In Tertiary Care Hospitals In Pakistan. Journal of Ayub Medical College Abbottabad. 2016;28(3):545–9. 28712232

[22] Kawado Miyuki, Hinotsu Shiro, Matsuyama Yutaka, Yamaguchi Takuhiro, Hashimoto Shuji, Ohashi Y. A comparison of error detection rates between the reading aloud method and the double data entry method Controlled Clinical Trials. 2003;24(5):560–9. doi: 10.1016/s0197-2456(03)00089-8 14500053

