# Supplementary material for: Assessing the knowledge and skill of vaccination staff at Adult Vaccination Counters for COVID-19 vaccines: Simulated client method
Source: PLoS One. 2021 Dec 23;16(12):e0261286. doi: 10.1371/journal.pone.0261286 (PMC8699954; doi:10.1371/journal.pone.0261286)
Supplement: S1 Questionnaire — (DOCX) [file pone.0261286.s001.docx]

**Questionnaire Scenarios**

For each AVC, the researcher will approach the AVC as a client who wishes to get the second dose and wants to get further information for his family. The researcher will act if he already received the first dose somewhere else but now he could not recall anything regarding the first dose and inquires for the second dose through the following scenarios. Additionally, the research will act if he also requires information for the first dose for his family member (spouse/sister/brother/father/mother). Any family member indicated should be cited to be above 18 years of age.

Note: The researcher must not take the second shot at all as the researcher’s second dose is actually due after 3 weeks. If the AVC has the mechanism to track the researcher’s first dose, then the researcher should continue seeking information for his supposed family member.

Scenarios for Question 2 in the questionnaire:

To determine that how many vaccines are available in the respective AVC, the respondents should be asked the following question(s):

1. Can I get to choose a vaccine of my choice?
   1. If yes, how many options do you have?
   2. If no, why? How many brands of vaccine do you have?

Scenarios for Question 3 in the questionnaire:

The purpose of the following scenarios is to see if the vaccination staff is able to track the client’s first vaccine dose.

1. I was vaccinated but don't recall the time or dose; could you please help check and administer the second dose?
2. I don't recall when I was vaccinated; could you kindly check and offer the second dose?
3. I do not recall the first dose name, could you please administer the second dose of the vaccine?
4. I don't recall when I was vaccinated; could you kindly check and offer the second dose?

Scenarios for Question 4 in the questionnaire:

To test knowledge of vaccination staff at each of the AVCs on different vaccine doses and durations, as well as to determine the practice of mix-matching vaccines, the following scenarios should be used. These scenarios address the general part of the Q.4 and it’s part b.

1. I want to get my [name the family member] vaccinated but I don’t trust certain brands. Could you please tell which vaccine brands do you have, so I can choose for them?
   - If the respondent tells that they have different brands of vaccine, ask for a specific vaccine?
2. I am interested in getting my [name the family member] vaccinated with Sino Zeneca vaccine (*deliberately calling incorrect vaccine name to check their knowledge of vaccines*). Can he/she get Sino Zeneca?
   - Please watch and wait if the respondent corrects you, and/or tells you about the different brands of vaccine and their availability at the AVC.
   - If the respondent doesn’t correct you, ask about the other brands of vaccine that may be available at their AVC.

*Scenarios for Question 4a in the questionnaire in case of “No” answer:*

1. If the vaccination staff could not recall the name of the vaccine, ask them that you would like to see the vaccine brand available so you could tell your family member about it.

*Scenario for Q.4c & d*

1. If my [name the family member] gets vaccinated with the vaccine [name the available vaccine(s) one by one], would she/he be done with the vaccination, right? I am unable to make multiple trips. *(Observe if the respondent tells you about vaccine doses and duration between the first and second dose)*.
2. If I am unable to return after four weeks, is it acceptable to administer the second dose after two weeks as I’ll be available then?
3. If CanSinoBio (requires only a single dose) is available at the AVC: After how many weeks after the first dose of CansinoBio should I bring my [name the family member] for the second dose?

*Scenario for Q.4e*

1. If the AVC does not have a certain vaccine brand, tell the respondent that you or your family member were inoculated with the unavailable vaccine brand but you want the second dose for yourself/your family member with the available vaccine brand at their AVC on your next visit.
   - For example, you find out that the concerned AVC did not have Sinopharm/Sinovac and was administering Astra Zeneca, tell, "I was given the first dose of Sinopharm and my second dose is overdue, can you please inject the second dose with Astra Zeneca?"
   - I had the first dose of Sinopharm, but I'd like to have a second dose of Astra Zeneca, as I've heard it's a wonderful vaccine. Could you please administer Astra Zeneca as the second dose?

Scenarios for Question 5 in the questionnaire:

1. Who is the focal person for COVID-19 vaccination in this AVC?
   - Are you a doctor sir?
   - I have few concerns. May I know your designation sir/ madam?

Scenarios for Question 6 in the questionnaire:

1. I am really afraid of needles. Some people have very good expertise to administer injections. Just curious, who will be administering the second dose of vaccine to me?

Scenarios for Question 7 in the questionnaire:

To determine if the other clients, who had been vaccinated, had received feedback on the second dose ask the following questions directly to the clients after they have existed the AVC. For this, wait outside of the AVC and portray that you want to get information as in client to client.

1. Have you been vaccinated?
2. Which vaccine was administered to you?
3. How many doses of the vaccine are there for the one administered to you?
4. When is the second dose due?
5. Do you have any information on how to obtain the vaccine certificate?

**Questionnaire to Fill the Details Obtained through Scenarios from the Vaccination Staff of Adult Vaccination Centers**

1. Name of health facility : _____________________________________________
2. How many types of vaccines do you have in your health facility?
   1. One b. Two c. Three d. Four e. _______
3. The hospital has a system to track the clients first dose Yes/ No
4. Is the hospital staff aware of the names of the vaccines that are available at their AVC? Yes/ No
   1. If no, play out the scenario so the vaccination staff show you the available vaccine.
   2. If yes, what brands of vaccines are available at the AVC? *(Instead of naming the vaccines, let them name it.)*
   3. How many doses of each brand of vaccine are required?
   4. If there are two doses for vaccination, after how much time the second dose is required?
   5. Can a different vaccine brand be inoculated than the brand of first vaccine dose? *(Inquire about the practice if vaccines doses are mixed/ paired it with others if same brand is not available on the second dose.)*

(Please input the above questions (b to e) in the following table):

|  | ***Vaccine Brands***  ***(a)*** | ***Availability***  ***(Y/N)***  ***(b)*** | ***No. of doses***  ***(c)*** | ***Second dose duration in days***  ***(d)*** | ***Knowledge of using different vaccine brand on the second dose than from the first dose***  ***(e)*** |
| --- | --- | --- | --- | --- | --- |
| *i.* | *Sinovac* |  |  |  |  |
| *ii.* | *Sinopharm* |  |  |  |  |
| *iii.* | *CanSino Bio* |  |  |  |  |
| *iv.* | *Astra Zeneca* |  |  |  |  |

1. What is the designation of the person who enters the vaccination record in to the digital system provided by the government?
   1. Medical Technician (MT)
   2. Nurse
   3. Lady Health Visitor (LHV)
   4. House Officer
   5. Other (please specify) _________________________
2. What is the designation of the person who administers the vaccine?
   1. Medical Technician (MT)
   2. Nurse
   3. Lady Health Visitor (LHV)
   4. House Officer
   5. Other (please specify) _________________________
3. Check with the last person who was vaccinated to see if they got the following details. Please make a circle.
   1. Name of vaccine _______________________
   2. Duration of second dose _______________________
   3. Information on how to obtain official vaccination certificate Yes / No
   4. Any other information _______________________
